# An account of the last autopsy carried out on a kuru patient

**DOI:** 10.1098/rstb.2008.4007

**Published:** 2008-11-27

**Authors:** Ken Boone

**Affiliations:** Goroka, c/o Papua New Guinea Institute of Medical Research PO Box 60, Goroka, EHP 441, Papua New Guinea

In my medical school, I learned of two diseases that Papua New Guinea was famous for among the medical profession throughout the world: pigbel and kuru. I was fortunate to be involved in the latter.

Over a conversation in Jerome Whitfield's apartment at Pacific Estate in Goroka one evening in 2003, I was asked whether I would be interested to be involved in the Kuru Project. Without hesitation I gladly accepted and that was the beginning of a new learning curve for me.

I was told that there was a known case of kuru who would be dying soon and that there was a need for someone to do a post-mortem out in the bush. A pathologist had been called in initially from London but had to leave when the patient had not died and time had run out for him. I was to do the job then. Consent for the autopsy had already been given by the family, as was described by Komit Poki during the conference (Poki 2008).

Later, one morning, I was told by my wife Lisa that Jerome had been calling me while I was out, and so on 7 April 2003 I left with Jerome on a Pacific helicopter to Ivingoi. That evening I went down with Jerome and Toby to see the patient. By then he was not talking. He had lost his gag reflex, had a bit of chest infection and had developed some bedsores. We dressed his sores and gave some medication to the relatives to help them nurse him and we left.

The next day we went to see the patient. I had a good look at all the instruments for the autopsy and familiarized myself with their use.

For the next 12 days we waited—until the evening of 20 April 2003, when word came that the patient had died.

At approximately 19.30 h, the three of us went in to perform the autopsy. It was dark by this time. We followed the planned safety procedures and got dressed in overall-type gowns that had hoods and boots. Our eyes and faces were protected by plastic screens, similar to the handheld glass used with a welding machine.

I wore a pair of gloves that had metal all woven together on the palm side. We were like astronauts from outer space.

After laying the body on an already prepared corrugated sheet table, I started the post-mortem.

I took specimens from every organ in the body. This included the whole brain, spinal cord down to the cauda equina, eyeballs, tonsils, thyroid, splanchnic vessels, the testis and a bit of peripheral nerve.

After doing the cutting, I would hand over the specimen to Jerome who would label it and pass it to Toby. He would then put it away in the iced boxes.

After working very hard during the night, I was asked to stop by Jerome at approximately 05.00 h.

The body was then sutured, washed and dressed nicely. A marble was inserted into the eyeball socket, the ‘smiling’ lips were glued and the body was laid into a coffin that had a glass window for the face.

All instruments, clothing, etc., were thoroughly cleaned with disinfectant and thrown into an already made deep pit. Petrol and kerosene were poured into it and burned. Following that the pit was covered and we left at approximately 06.15 h when the first people in the houses were coming out to relieve themselves.

At approximately 08.15 h, as soon as the weather was clear, a Pacific helicopter flew in to pick up Toby and me and the specimens. Jerome arrived back in Goroka later after sorting out a few things.

I cherished my time with the Kuru Project as I had been able to do a thorough and comprehensive post-mortem on one of the last confirmed cases of kuru. More than that, I found out that the specimens that I obtained are now being tested in animals in further experimental studies. This has made me happy as I feel I have contributed in one way or another to the development of basic scientific knowledge.

Finally, I look forward to being involved in the publications that will be written to describe the pathology found in the specimens I collected.
